# Treatment of kyphotic deformity in Kümmell’s disease through vertebral body screw fixation and intertransverse process grafting: A case report

**DOI:** 10.1097/MD.0000000000037058

**Published:** 2024-01-26

**Authors:** Hui Kang, Tanjun Wei, Wenbo Zeng, Shenghui Lan

**Affiliations:** a Department of Orthopaedics, General Hospital of Central Theater Command (Wuhan General Hospital of Guangzhou Command, previously), Wuhan, China; b Department of Orthopaedics, The Third Affiliated Hospital of Chongqing Medical University, Chongqing, China; c Department of Orthopaedics, The Eighth People’s Hospital, Jiangsu University, Shanghai, China.

**Keywords:** injured vertebral body screw fixation, intertransverse process grafting, Kümmell’s disease, kyphotic deformity

## Abstract

**Rationale:**

Kümmell’s disease, also well acknowledged as delayed posttraumatic vertebral body collapse, it is a rare condition which mainly occurs in elderly people more than 50 years old, with the thoracolumbar junction being mostly affected.

**Patient concerns:**

In this research, we employed posterior short-segment screw fixation within the injured vertebral region, coupled with intertransverse process bone grafting, to address Kümmell’s disease. A 57-year-old female was admitted to our institution with incapacitating back pain and obvious kyphotic deformity.

**Diagnoses:**

The diagnosis of Kummell disease was mainly depended on clinical symptoms and imaging examinations.

**Interventions:**

In this research, we employed posterior short-segment screw fixation within the injured vertebral region, coupled with intertransverse process bone grafting, to address Kümmell’s disease.

**Outcomes:**

The patient could walk independently with the help of a thoracolumbosacral orthosis brace on postoperative Day 2. No pains, kyphotic deformity and neurological deficits were observed during the 36 months of postoperative follow-up. These improvements can be visualized through postoperative magnetic resonance imaging and CT scans. Short-segment screw fixation provides short-term stability to the fracture site and accelerates fracture healing. Subsequently, the healed intervertebral and transverse process grafts offer long-term stability, a fact corroborated by postoperative CT scans.

**Lessons:**

In summary, for Kümmell’s disease patients exhibiting kyphotic deformity without neurological deficits or compression, posterior short-segment vertebral screw fixation with intertransverse process bone grafting stands as a viable alternative treatment approach.

## 1. Introduction

Kümmell’s disease, also well acknowledged as delayed posttraumatic vertebral body collapse, was firstly introduced by Kümmell in 1895.^[1]^ It is a rare condition which mainly occurs in elderly people more than 50 years old, with the thoracolumbar junction being mostly affected.^[2]^ With an aging population, the incidence of Kümmell’s disease is set to increase further soon. The incidence of Kümmell’s disease has been reported to vary from 12.1% to 42.4% in the osteoporotic vertebral compression fractures, with slight predominance in women because more osteoporotic vertebral compression fractures happen in women.^[3]^ The option of treatment for this disease is determined by several factors, including the patient comorbidities and symptoms, radiological findings, the severity of the spinal deformity and the presence of neurological deficits.^[4]^ Here we applied posterior approach using injured vertebral body pedicle screw and rod system fixation combined with intertransverse process grafting for 1 case of Kümmell’s disease with kyphotic deformity and reported our findings.

The most common affected segments are the thoracolumbar spine, mainly because of the dynamic differences between the relatively stiff thoracic segments and mobile lumbar segments.^[5]^ Generally, just 1 vertebra is involved, most frequently T11, T12 or L1.^[6]^ The remarkable characteristic clinical course of Kümmell’s disease is an asymptomatic interval following an initially trivial insult, at which time the initial examination and X-ray showed negative result. The asymptomatic interval may vary widely from several weeks to several months or even several years.^[1]^ Then the patient would suffer a gradually serious intensity of pain which would not respond to conservative treatment, and display delayed progressive vertebral collapse and intervertebral vacuum cleft consequently. In most cases, neurologic compromise rarely appears unless kyphosis or pseudoarthrosis occurs and the according spinal cord is compressed. So, it is commonly divided into 5 distinct continuous clinical stages, that is initial injury, post-traumatic period, latent interval, the recrudescent stage, and terminal stage.^[1]^

The treatments for Kümmell’s disease conclude conservative and surgical treatments. Conservative treatments including rest, orthosis, lumbar traction, antiosteoporotic therapies and painkillers gain controversy for a long time and should be considered at the early stage of the Kümmell’s disease or in patients with poor general conditions.^[7,8]^ Surgical managements, which can be divided into minimal invasive and open surgeries, should be considered whenever conservative treatments fail to improve the patient life qualities and prevent further deterioration of the patient symptoms. Minimal invasive surgeries consist of minimally invasive vertebroplasty, kyphoplasty and bone-filling mesh containers. Open surgeries, which consist of anterior approach and posterior approach, should be considered when neurological deficit or progressive kyphosis appear.^[9]^

## 2. Case report

**Figure 1.**
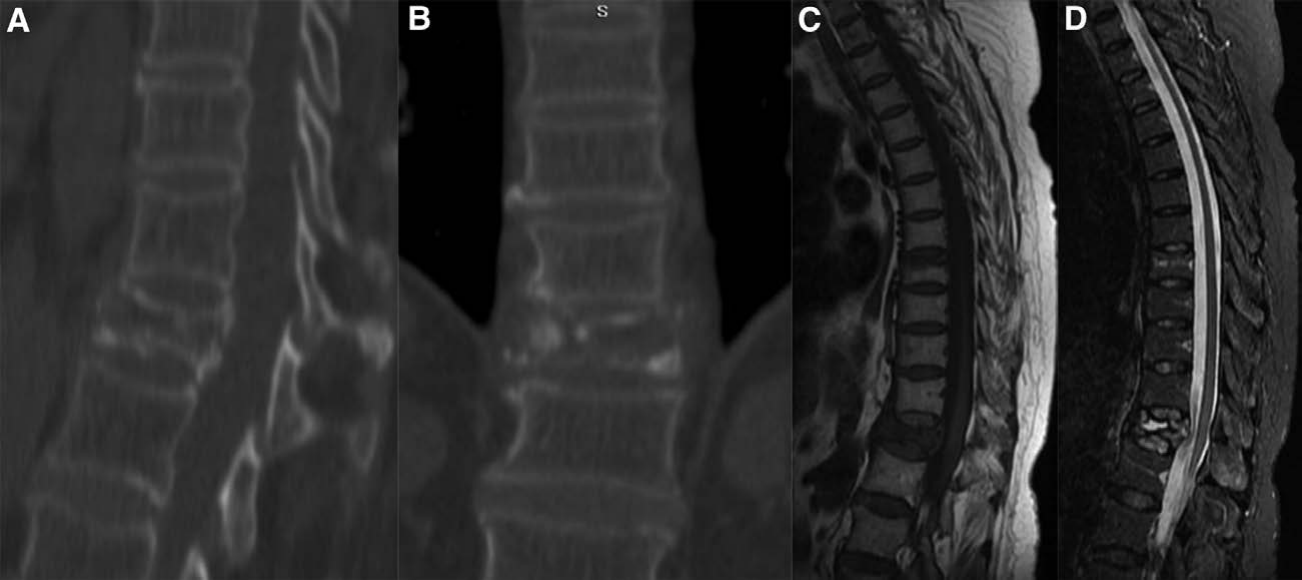
The images for T12 and T8 vertebral bodies. A, B: Axial (A) and coronal (B) CT scans confirmed the severe collapse together with a linear intervertebral vacuum cleft of T12 at the time of admission. C, D: Axial T1-weighted (C) and STIR (D) MR images showed an osteoporotic compression fracture of T8 and a linear intervertebral vacuum cleft in T12. CT = computer tomography.

**Figure 2.**
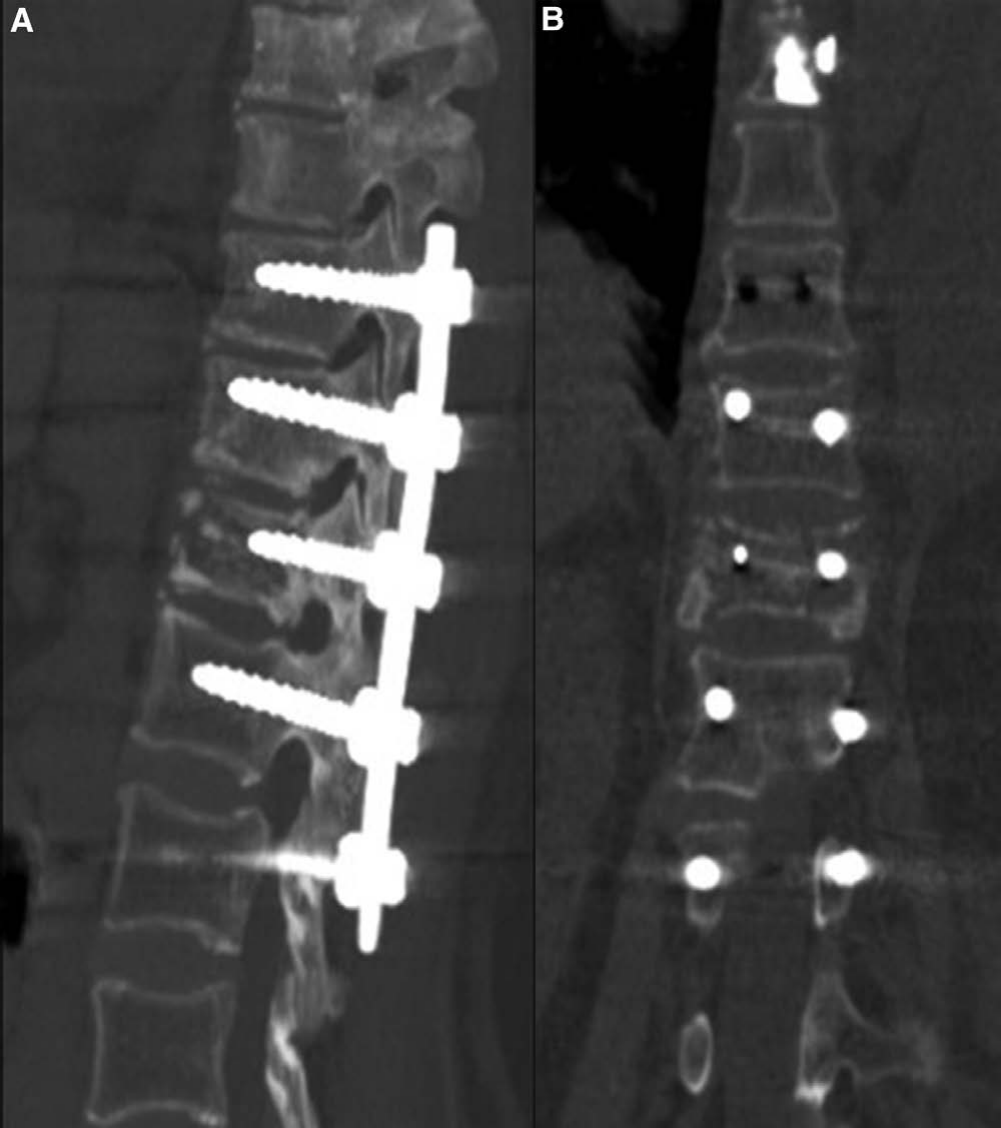
The images for T12 vertebral body treated by injured vertebral body screw fixation combined with intertransverse process grafting. A, B: Axial (A) and coronal (B) CT scans in T12 vertebral body. CT = computer tomography.

**Figure 3.**
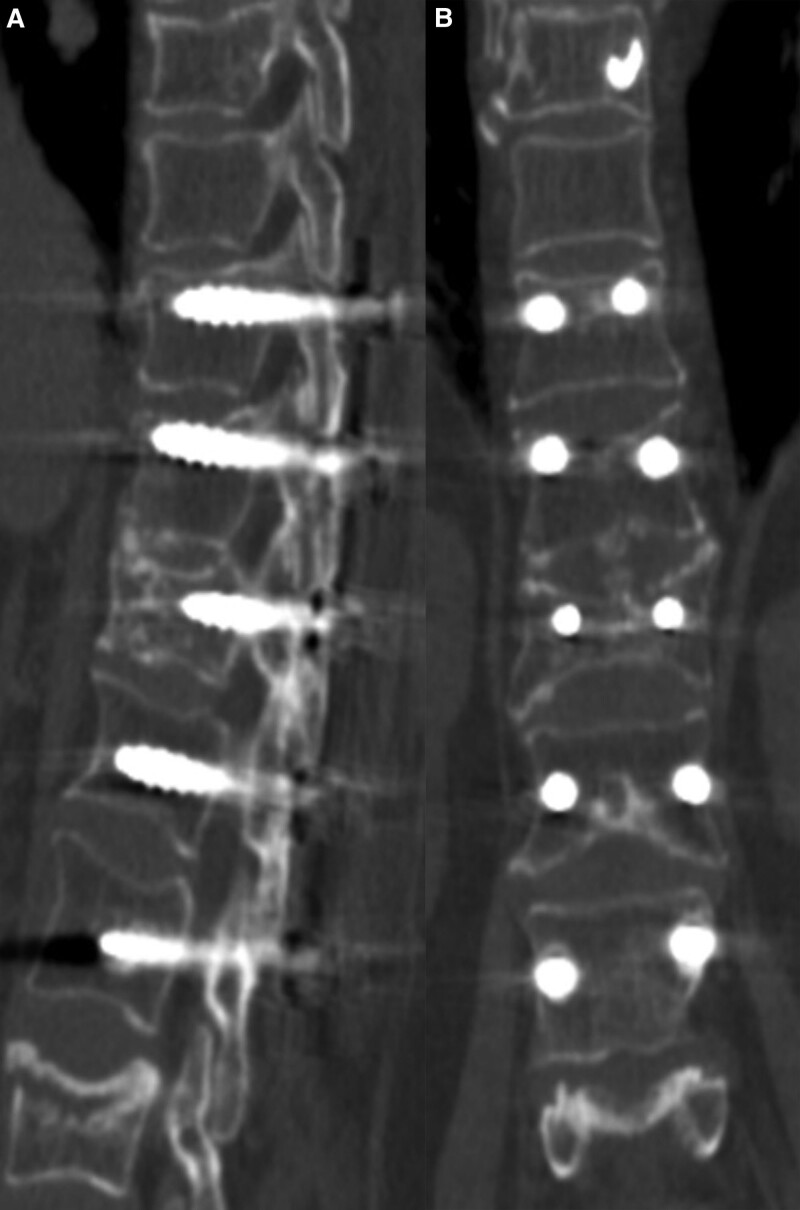
Good vertebral height, bone healing and new bone formation in T12 vertebral body. A, B: Axial (A) and coronal (B) CT scans in T12 vertebral body during the 36 months of postoperative follow-up. CT = computer tomography.

A 57-year-old female was admitted to our institution with incapacitating back pain and obvious kyphotic deformity. Three months before, the patient developed back pain after a slight lumbar strain. Radiographs were not obtained at that time and no special attention was paid for this mild injury. The patient had no symptoms until development of gradually aggravated back pains and kyphotic deformity. Standard radiographs revealed the severe compression fracture of T12 and a mild compression fracture of T8, as shown in Figure 1A and B. Physical examination showed percussion tenderness and localized axial pain on T8 and T12. No neurological deficits were noted. CT scan and 3-dimension reconstruction and magnetic resonance imaging clearly displayed a minimal collapse of T8 and a severe collapse together with a linear intervertebral vacuum cleft of T12, as shown in Figure 1C and D (CT scan and 3-dimension reconstruction of T8 was not shown because of image volume limitation). The intervertebral vacuum cleft showed decreased signal intensity on the T1 and increased signal intensity on T2-wighted images, as shown in Figure 1C and D. Laboratory testing and bone biopsy were performed to rule out any malignancies and infections. The patient was diagnosed with an osteoporotic compression fracture of T8 and Kümmell’s disease of T12. We performed posterior short-segment injured vertebral screw fixation combined with intertransverse process grafting for T12 to correct the kyphotic deformity and maintain stability (Fig. 2A and B), followed by a percutaneous vertebroplasty (PKP) for T8 after informed and written consent were obtained from the patient. The patient could walk independently with the help of a thoracolumbosacral orthosis brace on postoperative Day 2. No pains, kyphotic deformity and neurological deficits were observed during the 36 months of postoperative follow-up, as shown in Figure 3A and B.

## 3. Discussion

The treatment strategies for Kümmell’s disease should be based on patient general states and comorbidities, the clinical stage of disease, the radiologic findings, the pain intensity, the bone mineral density of the involved vertebra, the severities of the kyphotic deformity and the neurological symptoms.^[4]^ For patients with intact posterior vertebral wall while without neurological impairment, conservative treatments, including bed rest, osteoanabolic therapy, analgesic drugs as well as bracing, should be considered.^[10]^ When conservative treatments fail and the persistent pain still exists or kyphotic deformity appears, surgical managements should be considered. For the minimal invasive procedures, controversies exist about the superiority between PVP and PKP. The choice of surgical managements depends largely on the surgeon preference.

In this study, we used posterior short-segment injured vertebral screw fixation combined with intertransverse process grafting for the treatment of Kümmell’s disease. The rationality of injured vertebral body pedicel screw was based on the pathophysiological hypothesis of ischemic posttraumatic vertebral necrosis, intervertebral fibrocartilaginous membrane formation, post-traumatic osteolysis and impaired bone fracture healing process. Pedicle screw fixation of the injured vertebral body can destroy the fibrocartilaginous membrane, resect the sclerotic and necrotic area and restore the bone healing process, which could be seen in the magnetic resonance imaging and CT after the surgery. The short-segment screw fixation could provide short-term stability for the fracture site and promote bone fracture healing. Later, the healed vertebral body and intertransverse process grafting would provide the long-term stability, which was confirmed by the CT after the surgery. But one should bear in mind that this kind of management only could be applied to selected patients of Kümmell’s disease who did not have any neurological impairment and compression. Hence, we proposed that posterior short-segment injured vertebral screw fixation combined with intertransverse process grafting could be an alternative choice in selected patients.

In conclusion, the patients with delayed posttraumatic vertebral body collapse with kyphotic deformity (Kümmell’s disease) who had any neurological impairment and compression, posterior short-segment injured vertebral screw fixation combined with intertransverse process grafting could be an alternative.

## Author contributions

**Conceptualization:** Wenbo Zeng, Shenghui Lan.

**Data curation:** Hui Kang, Tanjun Wei.

**Formal analysis:** Hui Kang, Tanjun Wei.

**Funding acquisition:** Shenghui Lan.

**Investigation:** Hui Kang, Tanjun Wei, Wenbo Zeng, Shenghui Lan.

**Methodology:** Hui Kang, Tanjun Wei, Wenbo Zeng, Shenghui Lan.

**Project administration:** Wenbo Zeng, Shenghui Lan.

**Software:** Wenbo Zeng, Shenghui Lan.

**Validation:** Wenbo Zeng, Shenghui Lan.

**Visualization:** Wenbo Zeng, Shenghui Lan.

**Writing – original draft:** Hui Kang, Tanjun Wei.

**Writing – review & editing:** Wenbo Zeng, Shenghui Lan.
